# Hepatitis B Virus in Polish Blood Donors in the Period 2005–2019—Significant Changes in Epidemiology and Demographic Characteristics of Infected Donors

**DOI:** 10.3390/v17010060

**Published:** 2025-01-02

**Authors:** Aneta Kopacz, Dorota Kubicka-Russel, Grzegorz Liszewski, Ewa Sulkowska, Anna Chrzanowska, Paulina Zwolińska, Ewa Noceń, Anna Potępa, Magdalena Łętowska, Piotr Grabarczyk, the Group of Polish Blood Transfusion Centers for Blood Borne Pathogens

**Affiliations:** 1 Department of Virology, Institute of Hematology and Transfusion Medicine, 02-776 Warsaw, Poland; drussel@ihit.waw.pl (D.K.-R.); gliszewski@ihit.waw.pl (G.L.); esulkowska@ihit.waw.pl (E.S.); achrzanowska@ihit.waw.pl (A.C.); pzwolinska@ihit.waw.pl (P.Z.); enocen@ihit.waw.pl (E.N.); apotepa@ihit.waw.pl (A.P.); pgrabarczyk@ihit.waw.pl (P.G.); 2 Department of Transfusion Medicine, Institute of Hematology and Transfusion Medicine, 02-776 Warsaw, Poland; mletowska@ihit.waw.pl

**Keywords:** HBV, blood donor, incidence, prevalence

## Abstract

In the 1980s, Poland was a medium-endemic country, with one of the highest incidences of hepatitis B in Europe (45/10^5^ inhabitants). Pursuant to the WHO guidelines, obligatory vaccination was introduced in 1994–1996 (as a part of hepatitis B prophylaxis for newborns), and in 2000–2011, all 14-year-olds were vaccinated. To prevent transfusion-transmitted HBV infection (TT-HBV), since the 1970s, each donation has been tested for HBsAg and, since 2005, additionally for the presence of HBV DNA. Based on the data from the Blood Transfusion Centers, changes in HBV detection in Polish blood donors were analyzed, starting from the introduction of mandatory NAT screening until 2019. During the period under analysis, a total of 11,625 HBV-infected donors were identified: 97.95% were seropositive (confirmed HBsAg) and 2.05% were seronegative (NAT yields). The detection frequency for both categories of infections was significantly (*p* = 0.05) higher for men than for women (Residual Risk RR = 1.4 and RR = 2.63, respectively). Seropositive infections were detected more frequently (*p* < 0.05) in first-time donors than in repeat donors (RR = 360), while no significant differences were observed in the category of seronegative infections. A downward trend in HBsAg detection was observed in both first-time and repeat donors (Spearman’s coefficient R = −0.98 and R = −0.90, respectively). The frequency of HBsAg in first-time donors decreased 5-fold, and, in repeat donors, 30-fold. In both subpopulations, the largest decrease occurred in the age group ≤ 20 years (i.e., donors born between 1985 and 2001). The incidence of window period (WP) infections in the repeat donor group demonstrated a downward trend (R = −0.54, *p* < 0.05), and in the first-time donor group, no significant trend was recorded. For occult hepatitis B infection (OBI), no significant trend was observed in either donor subpopulation. WP infections were detected significantly more often in donors aged 21–50 years than in donors ≤20 years, most often in the 41–50 age group. The frequency of OBI increased with donor age and was the highest in the 51–60 age group. A spectacular decrease in the frequency of HBsAg(+) infections was observed in current study, indicating the effectiveness of the hepatitis prevention strategy applied in Poland. We expect that the improvement in the epidemiological situation among blood donors causes a reduction in the risk of TT-HBV. Confirmation of this hypothesis by the analysis of residual risk should be a subject of further studies.

## 1. Introduction

Hepatitis B virus (HBV) infections are one of the major worldwide epidemiological problems. According to WHO estimates, one-third of the world’s population have had contact with HBV, and almost 300 million are chronically infected. HBV causes acute, hyperacute and chronic hepatitis B, leading to the death of more than 800,000 people every year [1].

The virus is transmitted directly with blood, sperm and vaginal mucus and indirectly through contaminated instruments (medical, cosmetic, injectable, etc.). In high-endemic countries, infections occur mainly by the perinatal route and by exposure to blood among children up to 5 years of age, and in low-endemic countries by the horizontal route. The occurrence of infection in children up to 5 years of age results in the development of a chronic form in up to 95% of cases; in adults, in 5% [1].

The most effective way of preventing the spread of HBV is vaccination, which has been available since 1982 [2]. The WHO vaccination schedule recommended since 1991 includes three or four doses (Expanded Program on Immunization; EPI-HBV), especially starting on the first day after birth (fd-EPI-HBV) [1]. Immunization results in production of protective anti-HBs antibodies and long-term immune memory development [3,4,5]. The effectiveness of producing anti-HBs after all doses of EPI-HBV taken according to this schedule approximates 100%, but is lower (≤90%) in people first vaccinated after 18 years of age, in premature infants, obese persons, smokers and newborns of high-viremia mothers [6]. In countries where fd-EPI-HBV was introduced, the prevalence of HBsAg decreased by 64–100% after a dozen years or so [7]. Universal vaccination has been introduced in most countries of the world, with the exception of, e.g., some regions of Africa, Europe and the Mediterranean [8].

Other measures recommended by WHO, used in the prevention of HBV, include the sterilization of medical instruments, use of disposable medical equipment, identification and treatment of infected persons in screening actions, transfusion of blood and blood components and transplantation of organs and tissues only from donors without HBV infection markers [9].

Most countries in the world identify blood donors infected with HBV in mandatory screening (usually HBsAg and, in some countries, additionally HBV DNA and/or anti-HBc) [10].

Measures to eliminate HBV are still considered insufficient, as at least 1.5 million new infections are annually recorded worldwide [1]. In 2016, the WHO announced a program designed for the global elimination of HBV infections by 2030 through intensifying preventive actions and increasing the availability of screening tests, as well as diagnostics and treatment [8].

At the end of the 1980s, Poland was classified as a medium-endemic country (2–8% HBsAg), with one of the highest incidences of hepatitis (45/10^5^ inhabitants) in Europe. The spread of HBV was limited by the introduction of the mentioned WHO measures in medical institutions, vaccination of risk groups and compulsory vaccination (EPI-HBV in the newborn program with four doses: 0, 1, 2, 12 months from 1994 to 1999, and with three doses: 0, 1, 6 months from 2000, fd-EPI-HBV). Moreover, from 2000, obligatory vaccination of 14-year-olds (14y-EPI-HBV), who had not been vaccinated as newborn, was performed. In 2004, the percentage of the vaccinated population older than 18 years old (born before 1986) equaled 16%, while as many as 68-93% of teenagers 14–18 years old were obligatorily vaccinated in the 14y-EPI-HBV program. The percentage of people vaccinated as newborns increased from 52% in 1994, to 71% in 1995, and to more than 95% from 1996. In consequence, according to the epidemiological surveillance, in the 21st century, the incidence of hepatitis B fell below 1/10^5^ inhabitants, and the frequency of HBsAg below 2% (low-endemic country) [11,12]. In Poland, prophylactic actions in the blood transfusion service were implemented several years after the discovery of the virus—mandatory blood donor testing for HBsAg in 1972 and for HBV DNA in 2005 [9]. Systematic improvement in the epidemiology of HBV was demonstrated by the analyses of HBsAg detection in blood donors in the period 1995–2004, which was also confirmed by data from epidemiological surveillance [12,13].

The aim of our study was to analyze changes in the frequency of HBV infections in Polish blood donors after the introduction of mandatory HBV DNA screening in 2005. The analysis was based on the results of HBsAg and HBV DNA screening performed in the years 2005–2019 in first-time and repeat donors, taking into account seropositive (HBsAg positive) and seronegative (NAT yield—HBsAg-/DNA HBV+) infections and demographic factors (gender, age). The epidemiological situation was assessed for all regions of the country.

## 2. Materials and Methods

### 2.1. Blood Donors

Demographic data for donors and the results of HBV screening were reported by all 23 Blood Transfusion Centers (BTC), including 21 Regional Blood Transfusion Centers (RBTC), the Military Blood Transfusion Center and the Blood Transfusion Center of The Ministry of Internal Affairs and Administration. The blood transfusion service in Poland applies uniform criteria guidelines for the assessment of blood donor eligibility and the quality of the screening system. According to Polish law, only voluntary, unremunerated donors can donate blood and blood components [14]. For the previous year, all BTCs were obliged to report to the Insitute of Hematology and Transfusion Medicine (IHTM) the data on the number of donations and donors screened for infection markers, categorized according to sex and six age groups; ≤20, 21–30, 31–40, 41–50, 51–60 and >60. The analyses were performed for each BTC and, for all reported donors, divided into groups of first-time and repeat donors. According to current regulations, donors <18 and >65 years of age are allowed to donate blood only in special circumstances and, every time, the physician’s consent is required. Donors <18 years old additionally need a written legal guardian’s consent.

In the period 2005–2019, 17,746,969 donations were collected from 8,872,932 Polish blood donors and tested for HBsAg and HBV DNA. Of all donors, 67.4% were repeat donors and 32.6% were first-time donors. In the donor population, there were more men (72.5%) than women (27.5%). Detailed demographic characteristics of Polish blood donors were presented in a separate paper [15].

### 2.2. Methods of Screening and Verification

#### 2.2.1. HBsAg

In the years 2005–2019, HBs antigen screening was performed by enzyme-linked immunosorbent assays (EIA): Hepanostica Uniform II (BioMerioux, Lyon, France) or EIA System 3 (Ortho Clinical Diagnostics, Raritan, NJ, USA). Since 2007, EIA tests have been replaced by chemiluminescent tests: (CMIA) Architect HBsAg Qualitative or Quantitative (Abbott Diagnostics, Wiesbaden, Germany). In the period 2010–2017, some donations were tested with the Vitros HBsAg ES test (Ortho Clinical Diagnostics Inc., NJ, USA). Since 2017, the Monolisa HBsAg Ultra (Bio-Rad, Marnes-la-Coguette, France), Elecsys^®^ HBsAg II (Roche Diagnostics, Mannheim, Germany) and Alinity and HBsAg Qualitative II Reagent Kit (Abbott, Sligo, Ireland) have been successively introduced. A diagram presenting tests used in individual BTCs in the years 2005–2019 is included in the Supplementary Materials (Figure S1).

##### Serological Test Algorithms

If the result of the HBsAg test was reactive, the sample was marked as initial reactive (IR) and repeated twice with the same test. If both repeats were negative, the IR result was considered biological false reactive (BFR), and if HBV DNA was not detected in screening, the donation could be used for therapeutic purposes. If at least two out of three results were reactive, the sample was described as repeat reactive (RR); the donation could not then be used for clinical purposes, and the sample was referred for confirmatory tests, including a neutralization and/or HBV DNA testing. A positive neutralization test result and/or HBV DNA detection in HBsAg repeat reactive donation indicated HBV-seropositive infection (HBsAg+), (Figure S2 in the Supplemental Materials).

#### 2.2.2. DNA HBV

Nucleic acid testing (NAT) was performed in two alternative formats—with Polimerase chain reaction (PCR)-based tests in minipools (MP) of 6–24 plasma donations or with Transcription-Mediated Amplification (TMA) in individual donations (IDT). In the first year, there were no recommendations regarding the sensitivity of HBV DNA screening. Initially, the Ampliscreen HBV test (Roche Diagnostics, Mannheim, Germany) was performed in MP24. The 95% limit of detection (95% LOD) for this format was estimated for 120 IU/mL with respect to a single donation. High frequency of infections as compared to other countries was detected, and the effectiveness of detection of seronegative infections depended significantly on the sensitivity of NAT tests [13]; therefore, the limit of 24 IU/mL with respect to individual donations was introduced. In consequence, since 2007, the Ampliscreen test has been replaced by the Taqscreen MPX test (Roche Diagnostics, South Branchburg, NJ, USA), with 95% LOD estimated for 3.8 IU/mL, with the maximum number of donations in MP reduced to six. Until 2011, screening in MP6 was conducted with the Taqscreen MPX test, and later with the Taqscreen MPXv2 test (Roche Diagnostics, South Branchburg, NJ, USA). The 95% LOD calculated per single donation was assessed at 22.8 IU/mL and 13.8 IU/mL, respectively. Since the beginning of NAT application in blood donor screening, alternatively to MP, some of the BTCs performed HBV DNA testing in individual donations (IDT) using the TMA method. Until 2007, the screening was performed manually using the Procleix Ultrio (Gen-Probe Incorporated, San Diego, CA, USA) test with 95% LOD at 7.4 IU/mL and, in the years 2007–2009, on the Tigris System (Gen-Probe Incorporated, San Diego, CA, USA) with 95% LOD at 10.4 IU/mL. Since 2010, the sensitivity of the HBV DNA testing increased to 2.1 IU/mL after introducing Ultrio Plus tests (Novartis Diagnostics, Emeryville, CA, USA) on the Tigris, and later Ultrio Elite on the Panther System with 95% LOD assessed at 4.3 IU/mL (Grifols Diagnostics, Emeryville, CA, USA). The diagram presenting the use of test formats and tests in individual BTCs in the years 2005–2019 is included in the Supplementary Materials (Figure S3).

##### NAT Screening and Verification Algorithms

After obtaining a reactive result for a seronegative donation (HBsAg-) in a NAT screening, repeat and/or discrimination tests were performed in accordance with the applicable algorithm in BTC, followed by HBV DNA verification and additional tests (anti-HBc total and IgM and anti-HBs, ARCHITECT Anti-HBc test, Abbott, Sligo, Ireland and VIDAS Anti-HBs Total II test, BioMerieux, Lyon, France) at the Institute of Hematology and Transfusion Medicine (IHTM). A positive HBV DNA test at IHTM indicated the detection of a seronegative infection (NAT yield). Additional anti-HBc total, IgM and anti-HBs tests for NAT yield infections allowed to distinguish between the serological Window Period (WP) and the occult HBV infection (OBI). NAT yield infection was classified as I WP if no anti-HBc was detected in additional tests at IHTM and anti-HBs at a level differing by more than 10 IU/L from the previous donation and/or subsequent donor sample. It was classified as II WP if anti-HBc IgM was detected in the donation. OBI was indicated by the presence of anti-HBc or anti-HBs at a level differing by less than 10 IU/L from the previous donation and/or subsequent donor sample.

The algorithms of screening and confirmatory tests, as well as the NAT quality control system in force in the Polish blood transfusion service, have been described in detail in an earlier publication [16].

### 2.3. Definitions

First-time donors were defined as persons who donated blood for the first time in their lives in the reporting year, regardless of the number of donations made in the first year of being a blood donor.

The group of repeat donors included donors who also donated blood in the previous years [14].

### 2.4. Statistical Analysis

Data from BTCs were submitted to IHTM in the form of a completed uniform .xls sheet. The data were aggregated in Excel (Microsoft Office) and then analyzed in Statistica version 13.3 (Tibco, Palo Alto, CA, USA).

The frequency of HBV infected donors and donations was calculated per 10^5^ with 95% confidence intervals (95% CI). The frequency of HBsAg was calculated as the number of seropositive (numerator) divided by the number of screened donors (denominator). The frequency of persistant HBsAg (prevalence) was calculated as the number of seropositive first-time donors (numerator) divided by the number of first-time donors in the screening (denominator). The frequency of acute HBsAg(+) infection was calculated by dividing the number of repeat donors with HBsAg(+) infection (numerator) by the number of tested repeat donors (denominator). The frequency of WP infection was calculated as the number of donors with WP infection (numerator) divided by the number of donors subjected to NAT testing (denominator). The frequency of OBI was calculated by dividing the number of OBI detected in all donors (numerator) by the number of donors subjected to NAT screening (denominator).

Differences between the two frequencies were expressed as a relative risk (RR) with 95% CI. The significance of differences between two or more frequencies was assessed using a chi-square test. The differences were considered statistically significant at *p* < 0.05. The Spearman’s rank correlation coefficient was used to analyze the trend, with the calculation of the R coefficient and the significance of changes (*p* < 0.05).

The value R = 0 meant that the variables were not correlated. The value “0 < +R < +1” and *p* < 0.05 meant that the observed upward trend was statistically significant. The value of “−1 > R > 0” and *p* < 0.05 meant that the observed downward trend was statistically significant. No significant upward or downward trend was observed if the R values differed from those mentioned above and *p* < 0.05.

## 3. Results

### 3.1. HBV Epidemiology in Blood Donors in Poland (2005–2019)

A total of 11,625 HBV-infected donors were identified in Poland between 2005 and 2019: 97.95% of them were HBsAg positive and 2.05% seronegative, with HBV DNA (NAT yields). Both HBsAg(+) and NAT yield infections were significantly more frequently detected in men than in women (Table 1 and Table 2).

**Figure 1:**
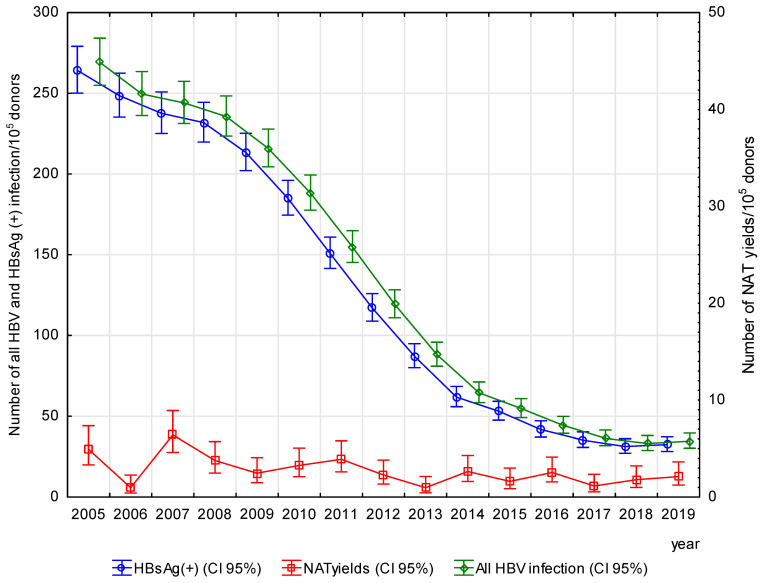
Frequency of HBV infection in total and stratified for seropositive donors and NAT yields—changes in Poland in the period 2005–2019.

The mean frequency of HBsAg(+) infection in all blood donors was 128.3/10^5^ donors and was 316-fold higher (*p* < 0.05) in first-time donors (391.04/10^5^ donors) than in repeat donors (1.24/10^5^ donors). Until 2018, the frequency of HBsAg(+) infections continued to decline from 264.3/10^5^ donors in 2005 to 31.4/10^5^ donors in 2018. Although the frequency of HBsAg(+) increased insignificantly in 2019 for the first time in the analyzed period as compared to the previous year (to 32.5/10^5^), a statistically significant downward trend was found for the entire period (R = −0.99, *p* < 0.05); Figure 1.

**Figure 2:**
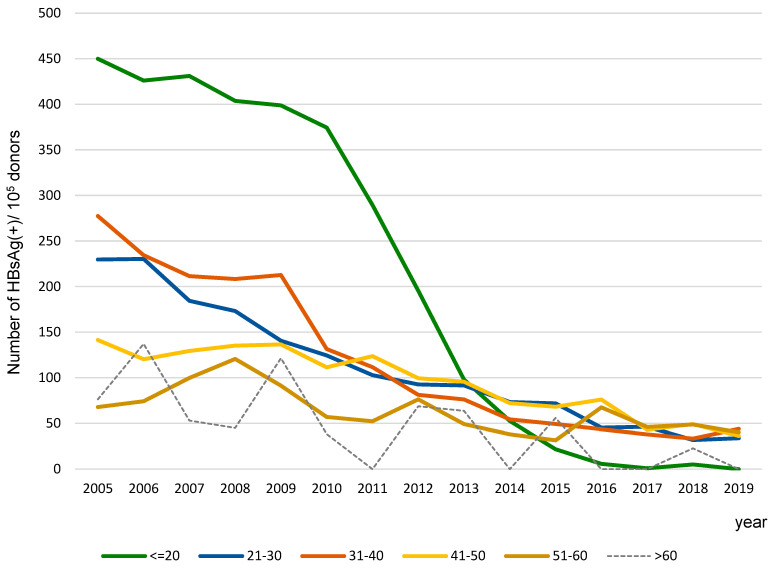
Frequency of HBV-seropositive infections in age groups—changes in Poland in the period 2005–2019. A significant decrease in the incidence of infections was observed in all age groups—correlation coefficient R > 0.67, *p* < 0.05. R for individual age groups (from the youngest) was: −0.99, −0.99, −0.97, −0.93, −0.74 and −0.68, respectively, *p* < 0.05 for all groups.

The data for the entire 2005–2019 period demonstrate that HBsAg(+) infections were most often detected in the youngest age group (224.5/10^5^ donors), and the least frequently in the oldest age group (35.68/10^5^ donors) (*p* < 0.05). The frequency of HBsAg(+) infections decreased with the donor age—it was significantly lower (*p* < 0.05) comparing each group to the youngest age group (≤20), and also when an older group was compared to a younger one, except the 31–40 to 21–30 age group (Table S1a). Over the 15-year period, there was a downward trend in the frequency of HBsAg(+) infections across all age groups. Significant changes between 2005 and 2019 occurred in all age groups except 51–60 (*p* = 0.16) and >60 years (*p* = 0.06). The largest (approximately 90-fold) decrease in the frequency of HBsAg(+) infections was observed in the youngest group of donors (*p* < 0.05), and a 2–7-fold in the rest of groups except >50 (*p* < 0.05) (Figure 2). Until 2012, the frequency of HBsAg(+) infections in the ≤20 years group of donors was the highest as compared to the other age groups (*p* < 0.05), but in 2015, it was already lower than in other age groups, except for the 51–60 age group, and remained the lowest beginning in 2016 (*p* < 0.05) (Figure 2).

#### 3.1.1. Epidemiology of HBV Seropositive Infections

**Figure 3:**
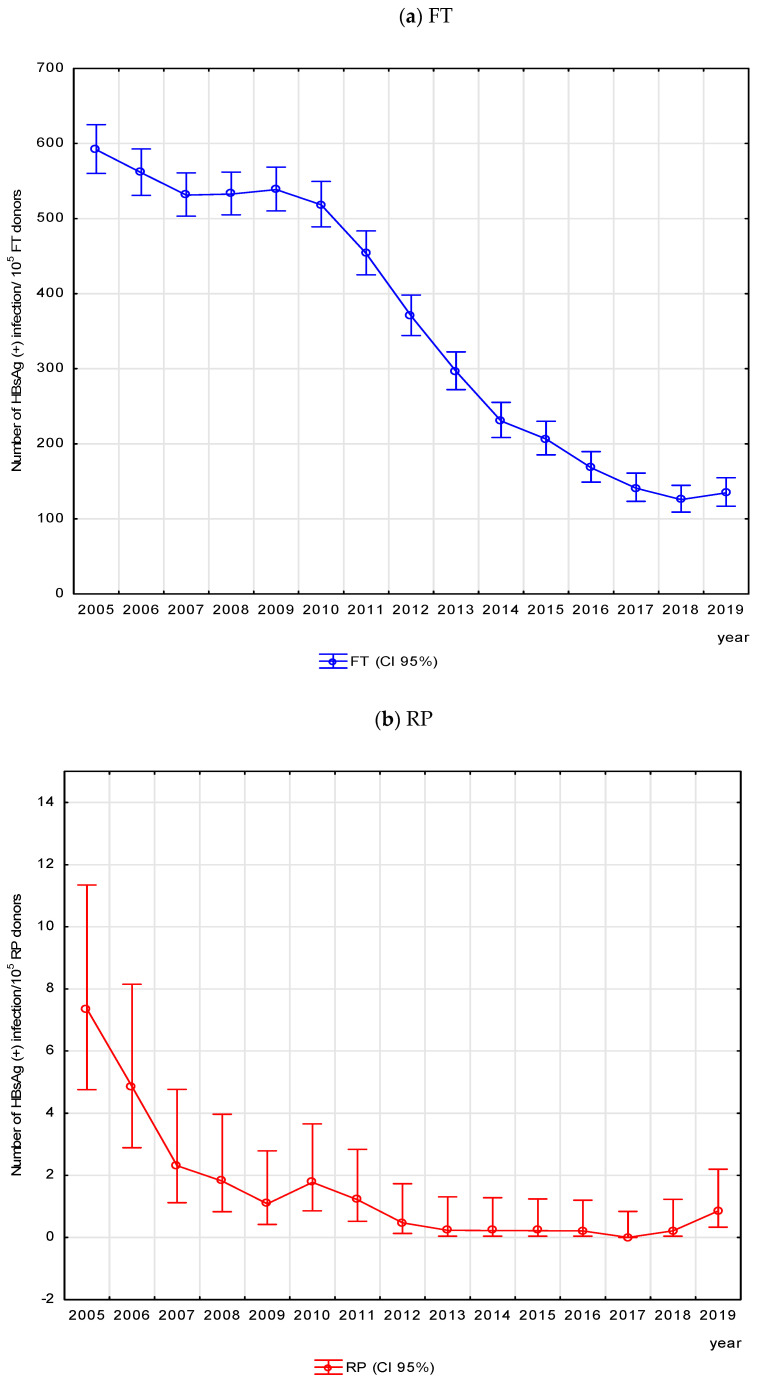
Frequency of HBV-seropositive infection in first-time donors (**a**) and repeat donors (**b**)—changes in Poland in the period 2005–2019.

The frequency of detected HBsAg(+) infections decreased approximately 5-fold in first-time donors (*p* < 0.05) and approximately 30-fold (*p* < 0.05) in repeat donors. In first-time donors, the highest (2.24-fold) reduction in the frequency of HBsAg(+) infections was observed in the period 2010-2014 (*p* < 0.05), and, in repeat donors, 3.18-fold in the years 2005–2007 (*p* < 0.05), (Figure 3).

**Figure 4:**
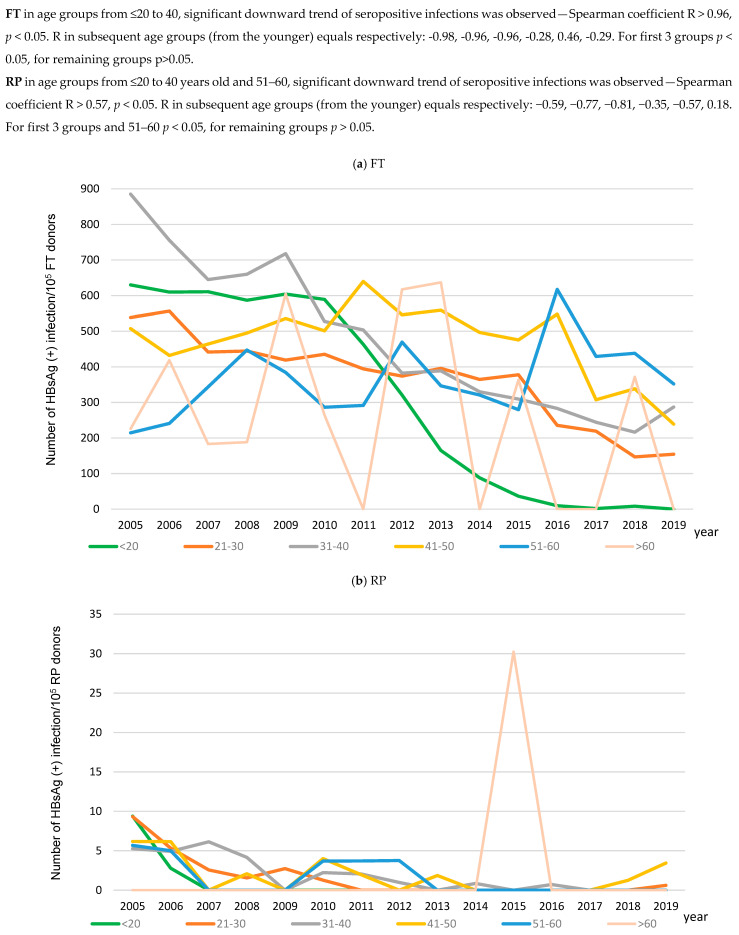
Changes of HBV-seropositive infections frequency in age groups, in Poland, in the period 2005–2019 in FT (**a**) and RP (**b**).

First-time donors, who were most frequently diagnosed with HBsAg(+) infections were in the 31–40 age group, and for repeat donors, it was the 41-50 age group (Table S1b,c). A significant decrease in the frequency of HBsAg(+) infections in first-time donors occurred in the three youngest age groups, and, in repeat donors, additionally in the 51–60 age group (Figure 4, Table S3).

#### 3.1.2. Epidemiology of HBV Seronegative Infections

**Figure 5:**
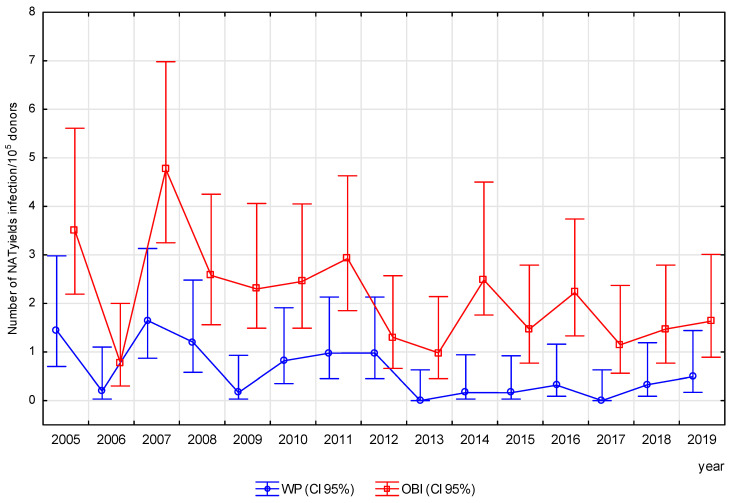
Frequency of HBV NAT yields—changes in Poland, in the period 2005–2019. In WP and OBI groups, no significant decreases in infection rates were observed—correlation coefficient for WP: R = −0.48, *p* > 0.05 and for OBI: R = −0.41, *p* > 0.05.

Out of the detected 238 NAT yield infections, 21.43% came from the serological window period and 78.57% from occult infection. The mean rate of NAT yield infection was 2.7/10^5^ donors, and, in this group, OBI was 3.6-fold more likely to be detected than WP (*p* < 0.05) (Table 2). The frequency of NAT yield infections decreased from 6.41/10^5^ donors in 2007 to 0.98/10^5^ donors in 2013 (RR = 6.54, *p* < 0.05), but there were no statistically significant trends over time either for WP or OBI (WP R = −0.49, *p* = 0.06; OBI R = −0.42, *p* = 0.12) (Figure 1 and Figure 5).

NAT yield infections were detected in all age groups of donors, and their frequency increased with donor age (p < 0.05 in relation of each subsequent age group to the youngest one and also when an older group was compared to a younger one, with the exception of 51–60 and >60 age groups) (Table S2a).

**Figure 6:**
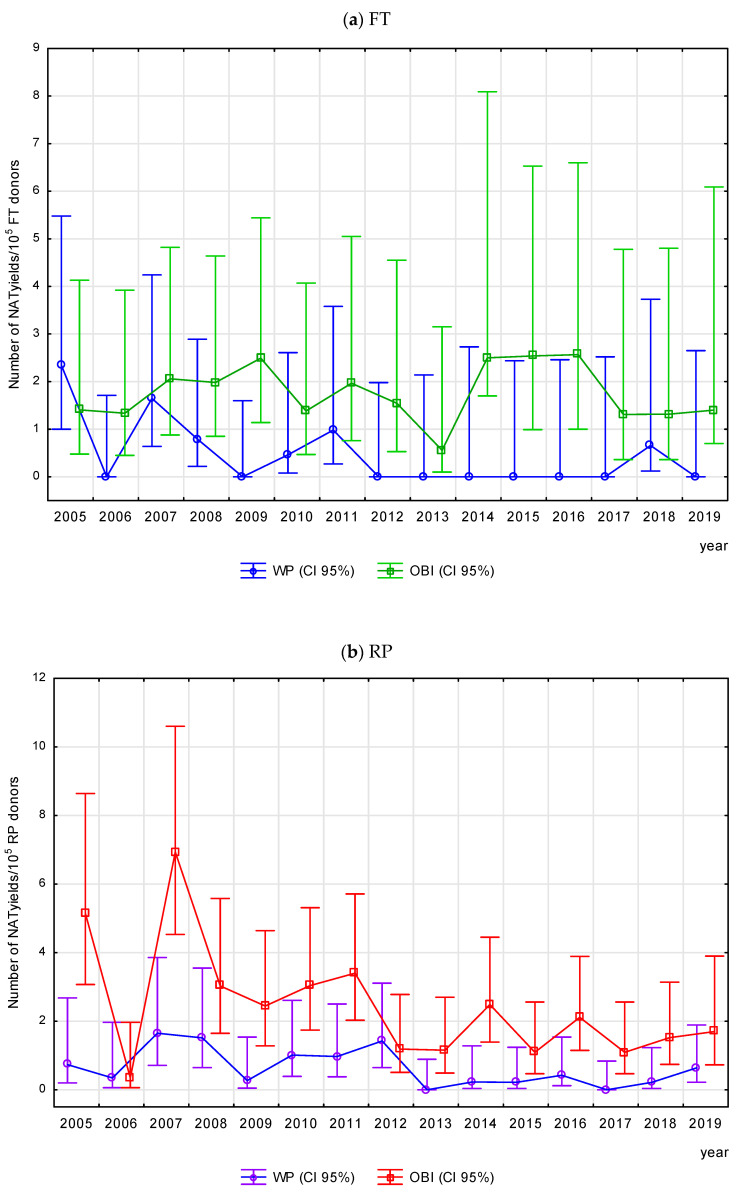
Frequency of WP and OBI in (**a**) first-time and (**b**) repeat donors—changes in Poland, in the period 2005–2019.

WP infections were detected significantly more frequently in the 21–50 age group than in the ≤20 age group, and most often (1.19/10^5^ donors) in the 41–50 age group (Table S2b). On the other hand, the frequency of OBI increased with donor age (*p* < 0.05), as demonstrated in the relation of each age group to the youngest one and in the relation of each age group (except > 60) to the next-younger group (*p* < 0.05), and was highest in the 51–60 age group (Table S2c). The incidence of WP infections in the repeat donor group ranged from 1.65/10^5^ in 2007 to 0.00/10^5^ donors in 2013 and 2017 and followed a downward trend (R = −0.54, *p* < 0.05). In the first-time donor group, it ranged from 2.34/10^5^ in 2005 to 0.00 in 2019 and demonstrated no significant trend (R = −0.50, *p* > 0.05). In contrast, the frequency of OBI infections in the repeat donor group ranged from 5.15/10^5^ in 2005 to 0.35/10^5^ in 2006; in the first-time donor group, it ranged from 2.57/10^5^ in 2016 to 0.56/10^5^ in 2013, and no significant trend was observed in either donor group (Figure 6a,b).

### 3.2. Frequency of HBV Infection in BTCs

**Figure 7:**
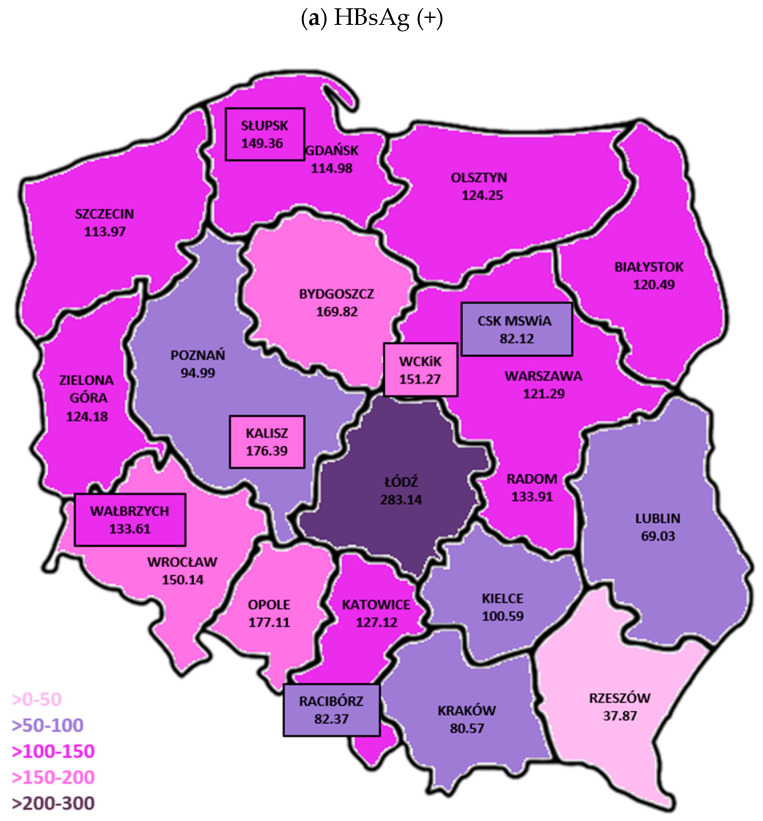

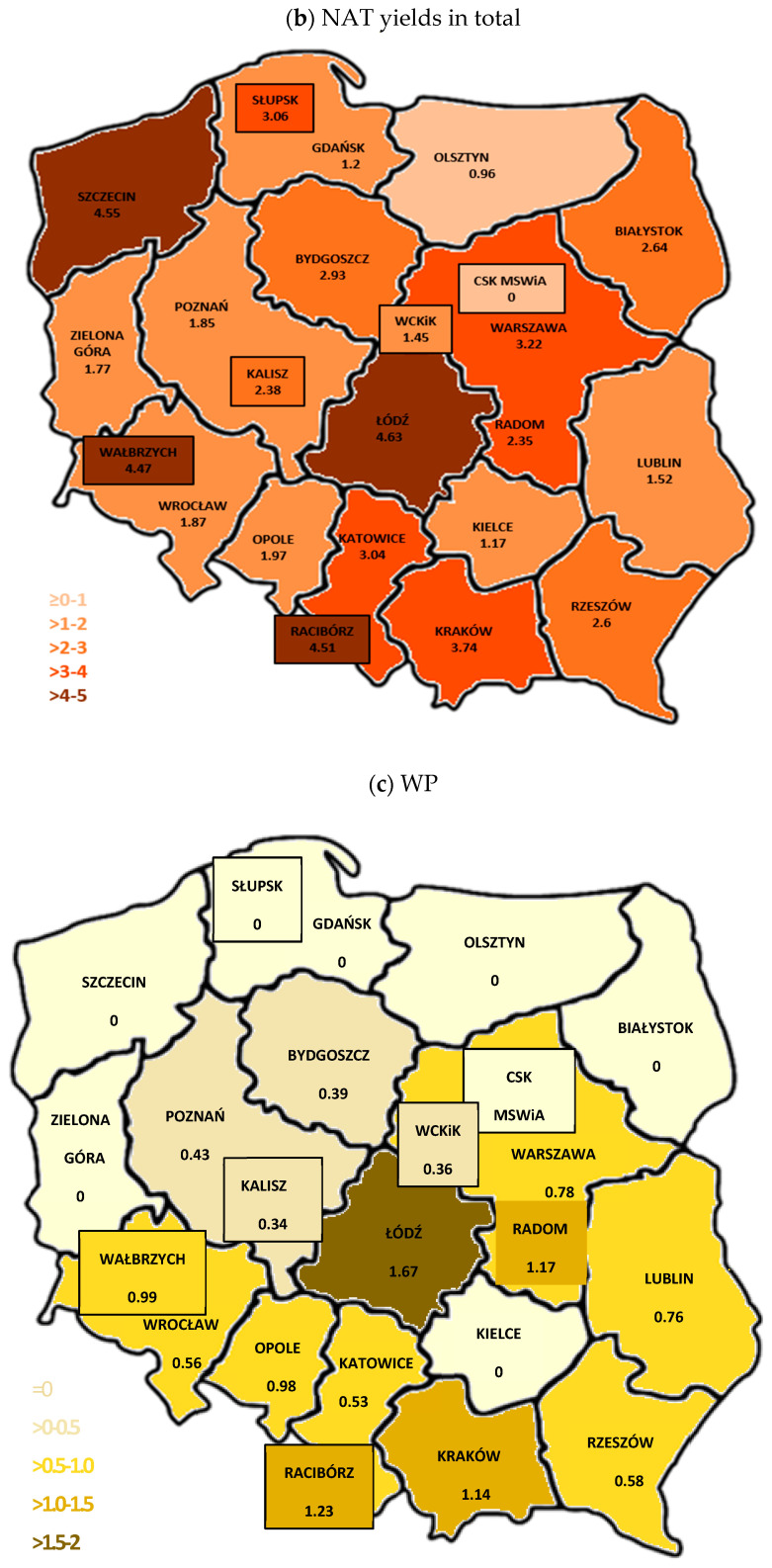

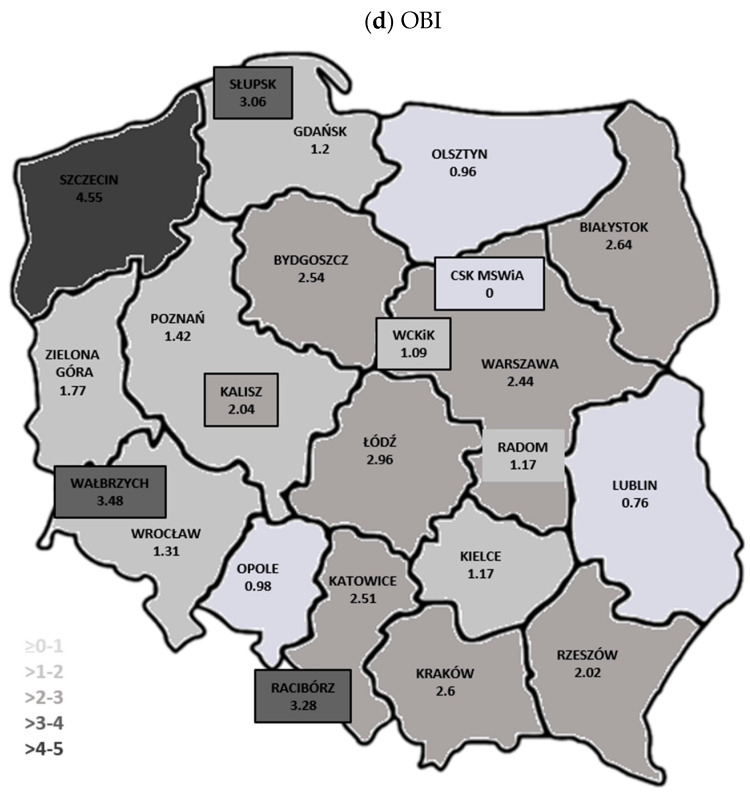
Frequency of HBV infection (number of HBsAg infection/10^5^ donors) in BTCs: (**a**) HBsAg(+), (**b**) NAT yields in total, (**c**) WP, (**d**) OBI.

The frequency of HBV infections differed between BTCs in different regions of Poland (Figure 7). The highest frequency of HBsAg(+) infections was reported in BTC Łódź (283.14/10^5^ donors), and the lowest in BTC Rzeszów (37.87/10^5^ donors). In four BTCs, the frequency of HBsAg was between 50 and 100/10^5^ donors; in 12 BTCs; between 100.1 and 149.99/10^5^ donors; and in five BTCs, it was between 150 and 200/10^5^ donors (Figure 7a). The frequency of NAT yields was the highest in BTC Łódź (4.63/10^5^ donors) and the lowest in BTC Olsztyn (0.96/10^5^ donors). In eight BTCs, the frequency of NAT yields was 1–2/10^5^ donors; in five, 2.1–2.99/10^5^ donors; and in seven, 3.0–4.55/10^5^ donors (Figure 7b). WP infections were detected in 15 out of 23 BTCs, most often—1.67/10^5^ donors—in BTC Łódź; in 3 BTCs, the frequency was between 1 and 1.23/10^5^; and in 11 BTCs, below 1/10^5^ donors (Figure 7c). On the other hand, OBI infections were detected in 22 out of 23 BTCs—a frequency below 1/10^5^ was observed in 3 BTCs, and in the rest of the BTCs, it was higher—in seven, between 1 and 1.99/10^5^ donors; in eight, 2.1–2.99/10^5^; and in 4 BTCs, 3–4.6/10^5^ donors (Figure 7d).

## 4. Discussion

Due to the limited availability of HBV screening data for the general population, analysis of the frequency of infections detected in blood donor screening are a valuable source of information from an epidemiological point of view. Screening tests for HBV markers in blood donors supports the implementation of WHO recommendations for the plan of worldwide elimination of hepatotropic infections by 2030 [8]. Our study indicates that in the 2005–2019 period, the average frequency of HBsAg(+) infection in Polish blood donors was 128.3/10^5^, and it was almost 3-fold lower as compared to the period 1995–2004, when the average frequency of seropositive infections approximated 349.7/10^5^ donors [13]. The currently observed frequency of HBsAg is lower than, e.g., Lithuania, South Africa and China [17,18,19], though still higher than in many Western European countries [20,21,22,23,24,25] and North America [26]. We believe that the high prevalence of HBsAg in Polish blood donors is the consequence of the HBV epidemic in 1980s [13]. It should be emphasized that the 2005–2019 period witnessed a dynamic and systematic decrease in HBV infections, especially HBsAg(+) in Polish blood donors: from 264.3/10^5^ in 2005 to 31.4/10^5^ in 2018 (Figure 1).

On average, the frequency of HBsAg(+) infections in Poland in the analyzed 15 years was 360-fold higher for first-time donors than for repeat donors. It should be emphasized that HBsAg frequency in FT donors represents prevalence, while in RP donors, it represents incidence. Over time, the difference between the frequency of HBV infection in first-time and repeat donors increased from 80.5-fold in 2005 to 496-fold in 2019, which is a continuation of the trend and its deepening from the period of the previous 10 years, when the difference between the respective groups of donors rose from 10-fold in 1995 to 50-fold in 2004 [13]. The increase in the difference between the frequency of seropositive infections in first-time and repeat donors in the period 1995–2004 was explaining by a decrease in the HBsAg rate of 20.7%/year in the repeat donor group as compared to only 5.4%/year in the first-time donor group. In the period 2005–2019, a higher dynamics of decrease in the frequency of HBsAg(+) infections was also observed in repeat donors as compared to first-time donors (RR for first vs. last year of observation was 30 and 5, respectively). Current and previous observations indicate a constant improvement in the epidemiological situation.

The main reason for the decline in the frequency of HBV infections in blood donors in 2005–2019, besides improvements in aseptic conditions in hospitals and medical institutions, was most likely attributed to vaccination against hepatitis B. From 1989 to 1993, vaccination was introduced in subsequent years for health employees with high risk of infections, infants born to HBsAg-positive mothers, other medical staff, students of medical colleges, healthy persons living with HBsAg carriers, patients with chronic diseases and in the preoperative period. The obligatory vaccination program for newborns started in some parts of Poland in 1994; in others, in 1995; and in the whole country, from 1996, leading to vaccination of 52%, 71%, 95% of newborns, respectively. Moreover, in the years 2000–2011, obligatory additional vaccination of 14-year-olds, who had not been vaccinated as newborns, was performed. The procedure was performed in 68–95% of this age group. The frequency of HBsAg declined in donors from all age groups, but changes were significant in the four youngest groups (≥18–50 years old, Figure S4). According to epidemiological surveillance reports, after the introduction of obligatory vaccination in the Polish population, the HBV incidence rate in general population decreased [12,27,28]. In blood donors, the impact of vaccination, especially fd-EPI-HBV, was manifested particularly by a 90-fold decrease in the frequency of HBsAg in donors from the youngest age group (born between 1985 and 2001). Of note, the youngest age group of donors in 2005, consisting of individuals born between 1985 and 1987, was mostly not vaccinated, while in 2019, most donors were obligatorily vaccinated as newborns. As shown in Figure 2, gradual decreases in HBsAg frequency in the youngest age group donor are the results of the entrance into the blood transfusion service of the population vaccinated in EPI-HBV programs: since 2005, in 14y-EPI-HBV; and from 2012, in fd-EPI-HBV (Figures S5–S7). Taking into account the above surveillance data, in the group of the youngest blood donors, respectively, from 2008 and 2016, the percentage of persons vaccinated at the age of 14 and as newborns increased to 96%.

Already in 2007, the impact of the higher percentage of vaccinated persons is recorded in the blood transfusion service as a significant decrease in the frequency of infections. It should be emphasized that the largest decrease in the frequency of HBsAg(+) infections in blood donors was observed in the years 2011–2013, when the youngest group of donors and no less that half of the donor group at 21–30 years were persons vaccinated at the age of 14. Moreovers from 2012s persons vaccinated as newborns started to enter the blood transfusion service (Figure 1, Figures S6 and S7). A significant decrease in HBsAg detection was also recorded in the years 2014–2016, when persons from the fd-EPI-HBV program joined the youngest donor group, and not less than 80% of the 21–30 age group were persons vaccinated at the age of 14. In the last 4 years of our analysis, the frequency of HBsAg is the lowest in the youngest group of donors, including almost exclusively individuals vaccinated as newborns, below 40/10^5^ in the groups 21–30 and 31–40 years (both included donors vaccinated as newborns or as 14y adolescents) and close to 50/10^5^ in older groups (not including donors vaccinated as newborns or 14y adolescents).

The decrease in the frequency of HBsAg(+) infections in repeat donors (30-fold) and infections detected in the serological window period (7.3-fold) illustrates the spectacular changes in the incidence of hepatitis B. Both HBsAg(+) and the window period infections in repeat donors were least frequently detected in the age group ≤ 20 years (0.87/10^5^ RP), comprising 15 out of 16 years of obligatory hepatitis vaccination (i.e., born in the years 1986–2001) and most often in the 41–50 age group (2.9/10^5^ RP), including no donors from the obligatory vaccination programs. Similar relationships were observed in Italy—in repeat blood donors, the lowest incidence was found in the youngest age group of vaccinated persons, but it was many-fold higher in age groups of persons who were not subjected to vaccination [25]. The epidemiological surveillance data are consistent with the results from the blood transfusion service and indicate a several-fold decrease in the frequency of acute hepatitis B (detected anti-HBc IgM) in Polish citizens. Between 2007 and 2019, the frequency of acute hepatitis B in the general population decreased from 0.95/10^5^ to 0.12/10^5^ [12,28]. In the period 2007–2018, the lowest frequency of acute hepatitis B (anti-HBc IgM+) was detected in persons subjected to EPI-HBV (from 0.06/10^5^ in 2007 to 0.05/10^5^ in 2019) and in vaccinated 14-year-olds (up to 0.27/10^5^ in 2007 and 2019). Acute hepatitis B was identified most frequently in 2007 in the 25–29 and >65 age group, and in 2019, in the 45–54 age group. Isolated cases of acute hepatitis B in children occurred due to lack of vaccination and lack of immunocompetence [12,28].

Data on HBsAg(+) infections in first-time donors illustrate the prevalence of HBV, which is usually the consequence of chronic infections. Among first-time donors, there was a 5-fold decrease in the frequency of HBsAg(+) infections, and the largest (600-fold!) was found in the youngest age group. Several observations indicate that HBsAg decreases were caused by the obligatory vaccination programs 14y-EPI-HBV and fd-EPI-HBV. In the youngest age group, the highest decline in HBsAg(+) infection was recorded in the period from 2011 (320.85/10^5^ donors) to 2016 (9.59/10^5^ donors), when the group initially consisted of donors vaccinated in 14y-EPI-HBV, and from 2011, the percentage of donors vaccinated in fd-EPI-HBV increased from 0% to 95%. From 2016 to 2019, when fd-EPI-HBV donors were mainly from the youngest group, HBsAg(+) frequency become the lowest (<10/10^5^ donors) of all donor groups (Figure 4a and Figure S6). The second-highest decline in HBsAg(+) frequency (3,5-fold) occurred between 2007–2019 in the 21–30 age group, when the percentage of donors vaccinated as 14-year-olds systematically increased (Figure 4a and Figure S7). The impact of obligatory vaccination is more evident in the period 2018–2019, when the group of ≤20-year-olds consisted mostly of persons mandatorily vaccinated in infancy, the 21–30 year-old group included persons vaccinated at 14 years old, the 31–40 year-old group partially included donors from the cohort vaccinated as 14y adolescents and donors at 41 to >65 years old did not include donors from the cohort vaccinated as newborns or 14y adolescents. The HBsAg frequency was 35–71-fold (*p* < 0.05) lower in donors vaccinated as newborns compared to those not immunized as newborns or 14y adolescents (Table S4b). HBsAg detection was significantly less frequent in the 21–30-year group (vaccinated at 14) than in the 31–40 group (partially vaccinated at 14y) and in the 41 to >65 year-group (not vaccinated) (Table S4, Figures S6 and S7). Epidemiological surveillance data for the period 2007–2019 indicate that for people aged 20–24, the frequency of HBsAg(+) infections (including chronic hepatitis) other than acute hepatitis decreased from 23/10^5^ in 2007 to 7.32/10^5^ in 2019 [13,28]. The largest, 15-fold, reduction in the frequency of chronic hepatitis B occurred in adolescents of 10–19 (covered with the vaccination of 14-year-olds or fd-EPI-HBV), and only isolated cases of chronic hepatitis B in children <10 years old subjected to fd-EPI-HBV occurred as the result of the lack of immunocompetence, vertical infections or contact with infected parents [13,27,28,29]. Similarly to Poland, several years after the implementation of obligatory hepatitis vaccination programs, Italy and South Africa, which belonged to areas of medium- and high-endemic prevalence of HBV, recorded at least a 5-fold decrease in the frequency of HBV infections in the youngest age groups of first-time donors [18,25]. According to analysis, a 3-fold decrease in the number of HBsAg(+) infections also occurred in the next 2 age groups of donors in both Poland and Italy [25]. This suggests that obligatory vaccination of newborns and 14-year-olds may also improve the epidemiological situation in other age groups not subjected to vaccination. There are also other factors that may have contributed to the reduction of infection rates, such as sterilization of medical instruments, the use of disposable medical equipment and the vaccination of high-risk groups (e.g., medics, infected pregnant women, cohabitants of infected persons). Probably such factors, other than being vacinated as a newborn or 14-year-old, improved the epidemiological situation among donors in the years 1997–2004, when a decrease in the frequency of HBsAg was observed: 1.2-fold in first-time donors and 2-fold in repeat donors, despite that there were no donors vaccinated in the obligatory fd-EPI-HBV program, and only in 2004 did the first 18-year-old donors begin to appear, who took part in the 14y-EPI-HBV program [13]. It is also worth noting that in a similar period, a 2–3-fold decrease in HBsAg frequency in first-time donors occurred in low-endemic countries,that have not introduced obligatory newborn or teenage vaccination, such as France and Switzerland [20,25]. The frequency of HBsAg(+) infections recorded in Poland in the analyzed period 2005–2019 is still higher than for Western European countries [20,23,24,25] and justifies the need to continue fd-EPI-HBV.

The high frequency of OBI infections, just as the previously discussed HBV seropositive infections, is the consequence of the reduced epidemic in the 1980’s. The hypothesis is supported by the observation of the increasing-with-age frequency of occult HBV infections in both first-time and repeat donors. Ye et al. demonstrated that in blood donors of subsequent age groups (<30, 31–40, 41–50, >50), the frequency of OBI and anti-HBc carriers increases significantly, due to a longer period of exposure to HBV [30]. Moreover, a characteristic feature of OBI infections is the ability to reactivate when immunity is reduced, e.g., as a result of immunosuppressive therapy [31,32,33]. The lowest frequency of OBI infections in blood donors from the youngest age groups allows for an expected reduction in the frequency of occult infection if vaccination and HBV prophylaxis will be continued. Such a forecast is significant from a public health point of view, as donations with occult HBV infection may cause hepatitis in the recipient, and the frequency of OBI in blood donors does affect the risk of TT-HBV [34]. Therefore, decreases in OBI rates can also result in a reduction in TT-HBV risk.

The presented analysis shows that the frequency of HBsAg detection among blood donors in different regions of Poland varies just as in the years 1995–2004. Like before, HBsAg(+) infections were most often identified in the BTCs Łódź, Kalisz and Opole, and least often in the BTCs located in Southeast Poland [13]. The frequency of NAT yields was also the highest in BTC Łódź, while the lowest frequency was observed in BTC Olsztyn and Gdańsk (Northeast Poland). As compared to the years 1995–2004, in the current analysis, the frequency of HBV infections in all BTCs was lower. A characteristic feature of OBI is low viral load, even <20 IU DNA HBV/mL [35]. It cannot be ruled out, that the NAT yield frequency in BTCs, e.g., Olsztyn and Gdańsk, may also be associated with lower sensitivity—as compared to other BTCs—of the NAT method (>20 IU DNA HBV/mL), which had been in use for at least half of the analyzed period.

Given the lower frequency of HBsAg-positive infections in repeat donors than in first-time donors (Table 1), an additional factor influencing the improvement of the epidemiological situation among blood donors could be a significant change in the ratio of repeat to first-time donors observed recently. Kubicka et al. demonstrated that in the period 2005–2018, the percentage of repeat donors, who, as it was presented in the current study are characterized by a lower frequency of HBV infections, increased by 19.83 percentage points [15]. In Poland, another possible factor that could reduce the HBV infection rate in blood donors was improvement in HBV screening. Adding NAT to HBsAg tests gives the possibility to detect more infected people (with low VL or seronegative). A donor with an detected infection was reported to the primary care physician with information about his infection. At the same time, the detection of HBV in the donor led to the destruction of the donation, thus preventing the transmission of infection during transfusions. Since 2017, questions have been added to the donor questionnaire, which may have contributed to the decrease in the frequency of detected HBV infections in donors. Affirmative answers to new questions, concerning events within 6 months before arrival for donation, such as cosmetic procedures that violate the skin layers, jaundice in a life/sexual partner, or close home stay/contact with a patient with viral hepatitis resulted in the disqualification of people at risk of infection and could reduce the number infections during screening.

Based on the presented results, similar to those of Lan Wei [36], strategies for the improvement of the epidemiological situation and reduction in the risk of TT-HBV may be suggested.The new policy of donor recruitment should focus more attention on the target donor population of women and repeat donors. The latter subpopulation of donors seems to be more aware of the HBV transmission routes and is therefore less affected by infections. The donor age-related increase in the frequency of HBV indicates the benefit of expanding the target population of young donors vaccinated against hepatitis.

### The Limitations of Our Study

Unfortunately, as presented in Materials and Methods, we have no data on the number of 18-, 19-, …, > 65-year-old donors (FT and RP) donating blood in BTCs each year (from 2005–2019). We have only total numbers of screened and infected donors from each of the six age groups. In consequence, we were not able to calculate for each year the precise proportion of donors vaccinated as adolescents at 14y and as newborns. Therefore, to interpret the impact of vaccination on the epidemiology of HBV infections among blood donors, we had to rely on vaccination schedules and coverage of the vaccination rate of the Polish population [11,37]. Moreover, we presented and discussed HBV frequency in FT and RP donors, which is important for risk assessment and transfusion safety. In this context, it should be noticed, that both FT and RP donors are screened in every donation; however, FT only once. Because the frequency of screening has an impact on the detection of seronegative HBV infection, WP or OBI frequency in FT and RP should not be directly compared. What is more, in WP and OBI, viremia is usually very low, so NAT yield detection increases with the sensitivity of the test [20,31,35]. In Poland, sensitivity of screening has increased, so in RP donors, who have donated for many years, the efficiency of low viremia detection of WP and OBI has also increased. This is the second argument against direct comparison of NAT yield frequencies between FT and RP donors. There are recent publications examining more effective immunity to HBV in females than in males, but because of the limited data, this issue in reference to Polish blood donors is yet to be analyzed. This we plan to do in our next publication.

According to the studies and assessments from other countries, it is known that the improvement in the epidemiological situation results is accompanied by the decreased risk of residual transmission of HBV infection [17,21,25]. The decrease in the frequency of HBV infections observed in the years 2005–2019 allows us to expect that the residual risk in Poland became lower and lower, taking into account also the higher and higher sensitivity of screening tests (Supplement Figure S3) [38]. The analysis of residual risk will be the subject of another publication, which could contribute important data to be considered in the debate on further optimization of screening algorithms for HBV in Poland.

## 5. Conclusions

The observed decrease in the incidence of HBV infections in blood donors in Poland in the years 2005–2019 was caused by the improvement in the epidemiological situation, which occurred due to the dissemination of measures to prevent the transmission of HBV during treatment procedures and vaccination against hepatitis B in risk groups (since 1989), 14-year-olds (2000–2011) and newborns (since 1994). The largest decrease in the frequency of HBsAg occurred in the youngest group of first-time donors, which was caused by the fact that the young people vaccinated as 14 years old teenagers or as newborns started to join the population of blood donors. Identification of HBV-infected blood donors during screening made it possible to refer them for treatment and apply appropriate prophylaxis for household members. At the same time, the detection of HBV in the donor led to the destruction of the infected donation, thus preventing the transmission of infection through transfusions. The higher incidence of HBV infections in blood donors in Poland compared to many European countries suggests that in order to increase blood safety in our country, it is necessary to continue newborn vaccination and to consider further improvement in donor recruitment and qualifications and strategies of donor screening.

## Figures and Tables

**Table 1:** Detection of HBV infection in Polish blood donors in 2005–2019 period in total. seropositive infections (HBsAg postive).

2005–2019	Donors	Tested	HBsAg Confirmed by Neutralization or DNA HBV Testing		
			No.	Frequency/10^5^ (CI 95%)	RR (CI 95%) *p*
DONORS	FT + RD	8,872,932	11,387	128.30 (126–130.7)	
	FTD	2,893,039	11,313	391.04 (383.9–398.3)	316 (251.4–397.2) *p* = 0.0000
	RD	5,979,893	74	1.24 (0.99–1.55)	
	Males *	6,434,700	8907	138.40 (135.6–141.3)	1.4 (1.35–1.48) *p* = 0.0000
	Females *	2,285,976	2241	98.03 (97.1–102.2)	
DONATIONS	FT + RD	17,746,969	11,387	64.20 (63–65.4)	
	FTD	2,893,039	11,313	391.04 (383.9–398.3)	785 (624.5–986.5) *p* = 0.000
	RD	14,853,930	74	0.50 (0.40–0.62)	

FT—first time donors, RD—repeat donors. * not data available from BTC Wałbrzych (for 2005–2015).

**Table 2:** Detection of HBV infection in Polish blood donors in 2005–2019 period in total. seronegative infections (NAT yields).

2005–2019	Donors	Tested	NAT Yields (in Total WP and OBI)			Window Period (WP)			Occult HBV (OBI)		
			No.	Frequency/10^5^ (CI 95%)	RR (CI 95%) *p*	No.	Frequency/10^5^ (CI 95%)	RR (CI 95%) *p*	No.	Frequency/10^5^ (CI 95%)	RR (CI 95%) *p*
DONORS	FT + RD	8,872,932	238	2.68 (2.36–3.05)		51	0.58 (0.44-0.76)		187	2.11 (1.83-2.43)	
	FTD	2,893,039	65	2.25 (1.76–2.86)	1.28 (0.96–1.71) *p* = 0.081	15	0.52 (0.31–0.86)	1.16 (0.64–2.12) *p* = 0.63	50	1.73 (1.31–2.28)	1.32 (0.95–1.83) *p* = 0.087
	RD	5,979,893	173	2.89 (2.49–3.36)		36	0.60 (0.43–0.83)		137	2.29 (1.94–2.71)	
	Males *	6,434,700	208	3.23 (2.82–3.70)	2.63 (1.77–3.91) *p* = 0.0000	43	0.67 (0.50–0.90)	2.55 (1.08–5.98) *p* = 0.026	165	2.56 (2.20–2.99)	2.66 (1.70–4.15) *p* = 0.0000
	Females *	2,285,976	28	1.22 (0.85–1.77)		6	0.26 (0.12–0.57)		22	0.96 (0.64–1.46)	
DONATIONS	FT + RD	17,746,969	238	1.34 (1.18–1.52)		51	0.29 (0.22–0.38)		187	1.05 (0.91–1.22)	
	FT	2,893,039	63	2.18 (1.70–2.79)	1.84 (1.38–2.46)	13	0.45 (0.26–0.77)	1.76 (0.94–3.30)	50	1.73 (1.31–2.28)	1.87 (1.35–2.59)
	RD	14,853,930	175	1.18 (1.02–1.37)		38	0.26 (0.19–0.35)		137	0.92 (0.78–1.09)	

FT—first time donors, RD—repeat donors. * not data available from BTC Wałbrzych (for 2005–2015).

## Data Availability

Data are contained within the article.

## Supplementary Materials

The following supporting information can be downloaded at: https://www.mdpi.com/article/10.3390/v17010060/s1: Figure S1. Methods of HBsAg screening used in Polish blood transfusion service (2005–2019); Figure S2. Algorithm of HBsAg confirmation; Figure S3. Methods of NAT screening used in Polish blood transfusion service (2005–2019); Figure S4. Changes of HBV seropositive infections frequency (/1000 donors) in age groups, in Poland, in the period 2005–2019 in FT and RP; Figure S5. Change (%) in the age structure of FT donors [15]; Figure S6. Fraction (%) of adult residents in Poland vaccinated as a newborn, changes in 2012–2019 [11]. Figure S7. Fraction (%) of adult residents in Poland vaccinated as 14-year-old adolescence, changes in 2005–2019 [37]; Table S1. Frequency of seropositive HBV (HBsAg+) infections in donor age groups in total, in Poland, in 2005–2019; Table S2. Frequency of HBV NAT yields in donors age groups in total, in Poland, in 2005–2019; Table S3. Spearman correlation (0 > R > 1 and *p* < 0.05 trend increase, −1 > R > 0 and *p* < 0.05 trend decrease) in age groups of seropositive donors.; Table S4. Frequency of seropositive HBV (HBsAg+) infections in donor age groups in total, in Poland, in 2018–2019.
