# Supplementary material for: Hepatitis B Virus in Polish Blood Donors in the Period 2005–2019—Significant Changes in Epidemiology and Demographic Characteristics of Infected Donors
Source: Viruses. 2025 Jan 2;17(1):60. doi: 10.3390/v17010060 (PMC11768870; doi:10.3390/v17010060)

Figure S1. Methods of HBsAg screening used in Polish blood transfusion service (2005-2019).

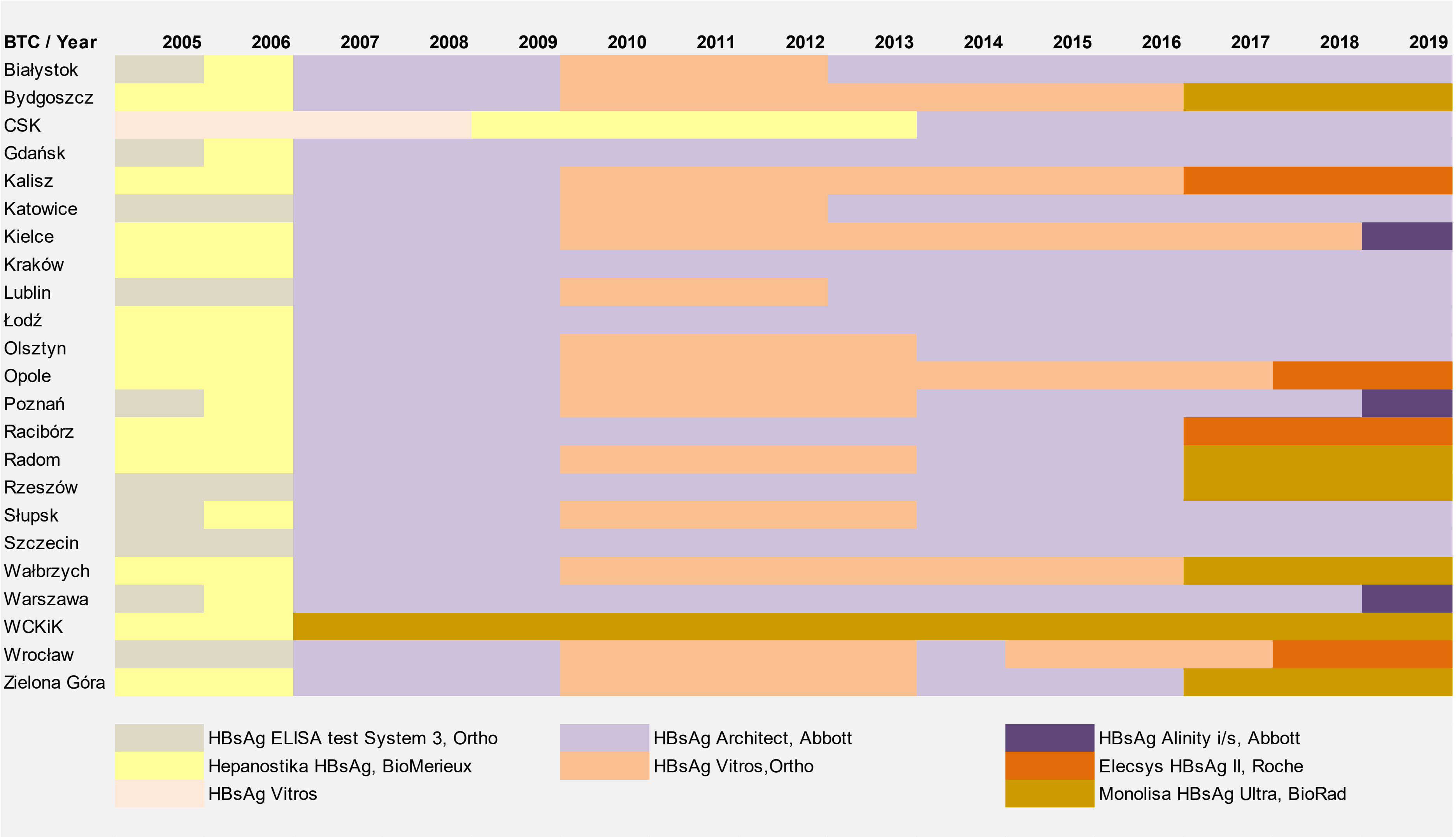

Figure S2. Algorithm of HBsAg confirmation.

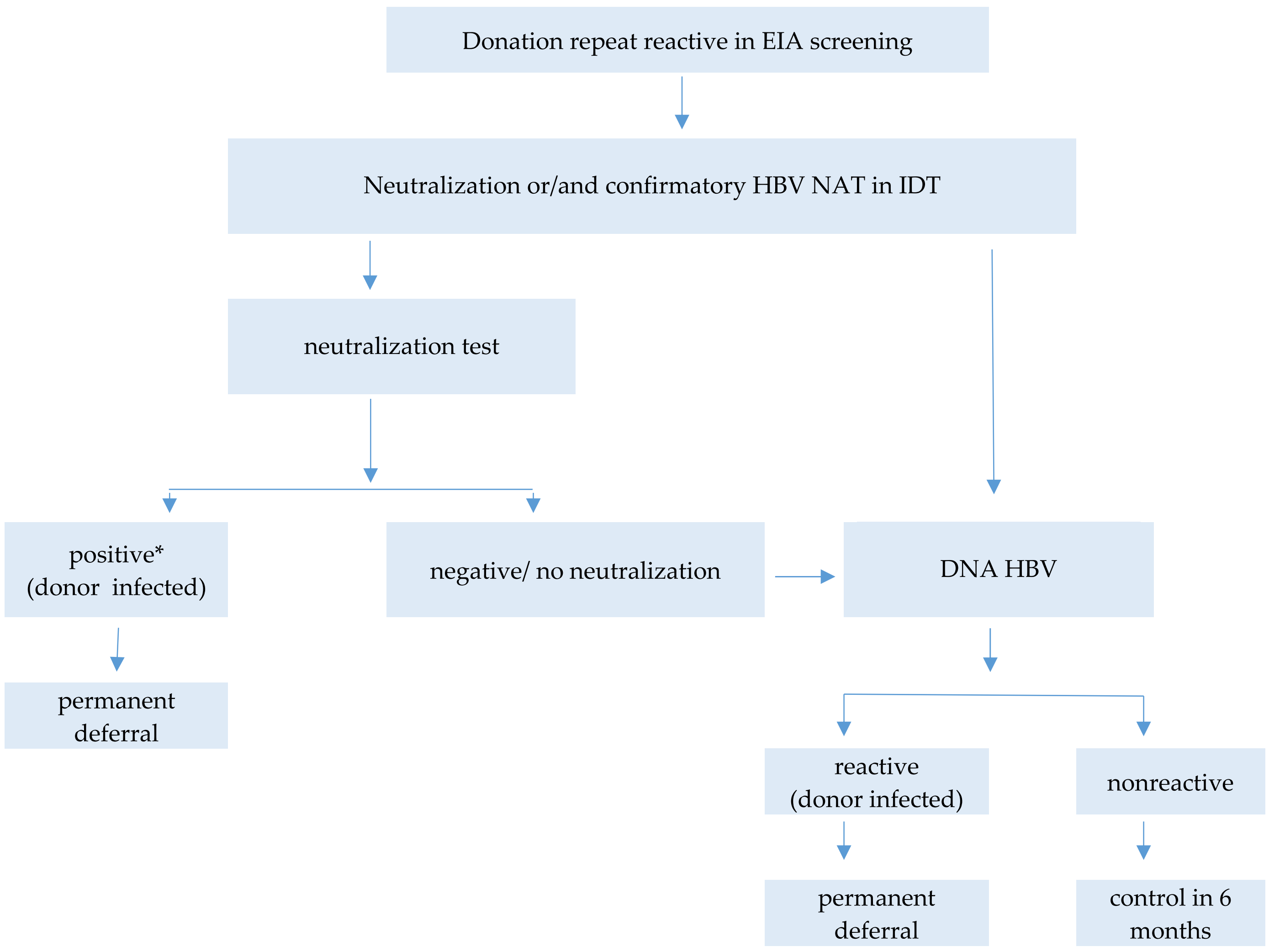

*if donor was not vaccinated in last 3 weeks before donation

Figure S3. Methods of NAT screening used in Polish blood transfusion service (2005-2019).

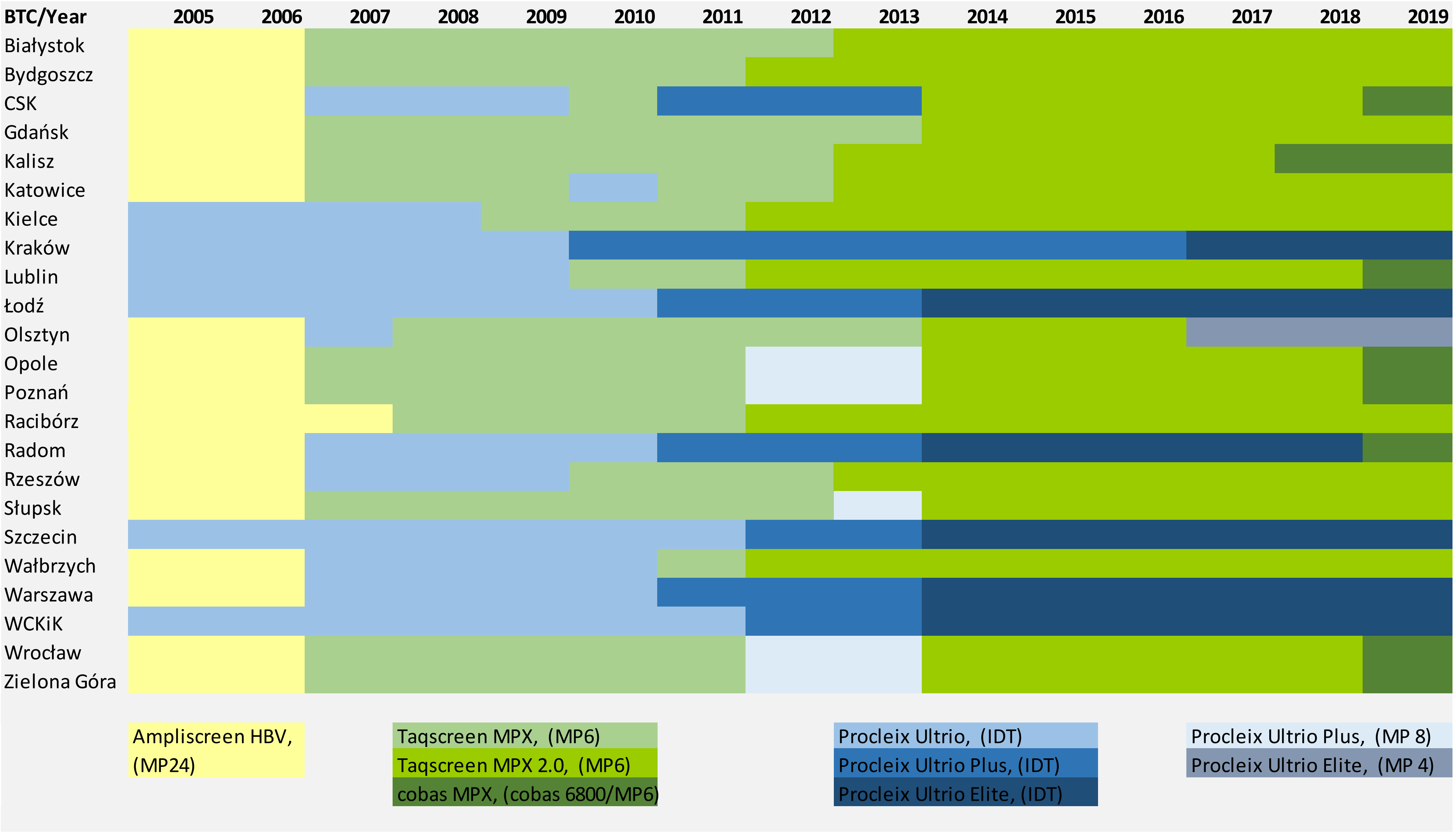

Table S1. Frequency of seropositive HBV (HBsAg+) infections in donor age groups in total, in Poland, in 2005-2019.

Year of birth	Age group	*No.* tested	*No.* HBsAg(+)	Frequency/10⁵ (95% CI)	Relative Risk (RR) between age groups: RR (95% CI) *younger group as a reference group*	p	RR (95% CI) *youngest group as a reference group*	p
a) In total								
2001-85	*<=20*	1,919,291	4,305	224.30 (217.71-231.09)	-	-	-	-
1998-75	*21-30*	3,224,788	3,503	108.63 (105.09-112.28)	0.48 (0.46-0.50)	0.0000	0.48 (0.46-0.50)	0.0000
1988-65	*31-40*	1,972,621	2,035	103.16 (98.78-107.74)	0.94 (0.89-1.00)	0.0641	0.45 (0.43-0.48)	0.0000
1979-55	*41-50*	1,088,745	994	91.30 (85.80-97.15)	0.88 (0.82-0.95)	0.0016	0.40 (0.37-0.43)	0.0000
1969-45	*51-60*	454,686	296	65.1 (58.10-72.96)	0.71 (0.62-0.81)	0.0000	0.29 (0.25-0.32)	0.0000
<1945	*>60*	44,869	16	35.66 (21.95-47.92)	0.54 (0.33-0.90)	0.0173	0.15 (0.09-0.25)	0.0000
b) First time donors								
2001-85	*<=20*	1,122,129	4,295	382.75 (371.50-394.35)	-	-	-	-
1998-75	*21-30*	891,089	3,469	389.30 (376.56-402.44)	1.02 (0.97-1.06)	0.4585	1.02 (0.97-1.06)	0.4585
1988-65	*31-40*	412,649	2,010	487.10 (466.31-508.81)	1.25 (1.18-1.32)	0.0000	1.27 (1.20-1.34)	0.0000
1979-55	*41-50*	207,962	977	469.80 (441.31-500.12)	0.96 (0.89-1.04)	0.3549	1.22 (1.14-1.31)	0.0000
1969-45	*51-60*	82,571	290	351.21 (313.11-393.94)	0.74 (0.65-0.85)	0.0000	0.91 (0.81-1.03)	0.1570
<1945	*>60*	5,439	15	275.79 (167.21-454.56)	0.78 (0.47-1.32)	0.3607	0.72 (0.43-1.19)	0.2037
c) Repeat blood donors								
2001-85	*<=20*	692,959	4	0.58 (0.22-1.48)	-	-	-	-
1998-75	*21-30*	2,329,652	28	1.20 (0.83-1.74)	2.1 (0.73-5.9)	0.1606	2.1 (0.73-5.9)	0.1606
1988-65	*31-40*	1,558,043	20	1.28 (0.83-1.98)	1.06 (0.60-1.89)	0.8221	2.22 (0.76-6.51)	0.1341
1979-55	*41-50*	879,772	15	1.70 (1.03-2.81)	1.32 (0.68-2.59)	0.4044	2.95 (0.98-8.89)	0.0434
1969-45	*51-60*	371,535	5	1.35 (0.57-3.15)	0.78 (0.28-2.17)	0.6461	2.33 (0.62-8.68)	0.1937
<1945	*>60*	39,399	1	2.54 (0.45-14.38)	1.88 (0.22-16.14)	0.5559	4.40 (0.49-39.3)	0.1474

Table S2. Frequency of HBV NAT yields in donors age groups in total, in Poland, in 2005-2019.

Year of birth	Age group	*No.* tested	*No.* NATyields	Frequency/10^5 (95% CI)	Relative Risk (RR) between age groups: **RR** (95% CI) ***younger*** *group as a reference group*	**p**	**RR** (95% CI) ***youngest*** *group as a reference group*	**p**
a) NAT yields (FT+RP)								
2001-85	*<=20*	1,919,291	5	0.26 (0.11-0.61)	-	-	-	-
1998-75	*21-30*	3,224,788	35	1.09 (0.78-1.51)	4.16 (1.63-10.63)	0.0012	4.16 (1.63-10.63)	0.0012
1988-65	*31-40*	1,972,621	40	2.03 (1.49-2.46)	1.86 (1.18-2.94)	0.0061	7.78 (3.07-19.72)	0.0000
1979-55	*41-50*	1,088,745	75	6.89 (5.50-8.63)	3.39 (2.31-4.98)	0.0000	26.44 (10.69-65.38)	0.0000
1969-45	*51-60*	454,686	74	16.27 (12.97-20.43)	2.36 (1.71-3.25)	0.0000	62.47 (25.25-154.52)	0.0000
<1945	*>60*	44,869	9	20.6 (10.55-38.12)	1.23 (0.61-2.46)	0.5531	76.99 (25.80-229.73)	0.0000
b) WP (FT+RP)								
2001-85	*<=20*	1,919,291	3	0.16 (0.05-0.46)	-	-	-	-
1998-75	*21-30*	3,224,788	21	0.65 (0.43-1.00)	4.16 (1.24-13.96)	0.0120	4.16 (1.24-13.96	0.0120
1988-65	*31-40*	1,972,621	12	0.61 (0.35-1.06)	0.93 (0.45-1.89)	0.8507	5.82 (1.28-26.57)	0.0232
1979-55	*41-50*	1,088,745	13	1.19 (0.70-2.04)	1.96 (0.89-4.30)	0.0860	11.36 (2.52-51.25)	0.0002
1969-45	*51-60*	454,686	2	0.44 (0.12-1.60)	0.36 (0.08-1.63)	0.1707	4.44 (0.63-31.49)	0.2362
<1945	*>60*	44,869	0	0.00 (0.00-8.56)	0	0.6569	0	0.7911
c) OBI (FT+RP)								
2001-85	*<=20*	1,919,291	2	0.10 (0.03-0.38)	-	-	-	-
1998-75	*21-30*	3,224,788	14	0.43 (0.26-0.73)	4.16 (0.94-18.33)	0.0402	4.16 (0.94-18.33)	0.0402
1988-65	*31-40*	1,972,621	28	1.42 (0.98-2.05)	3.26 (1.72-6.21)	0.0001	13.6 (3.24-57.17)	0.0000
1979-55	*41-50*	1,088,745	62	5.69 (4.44-7.30)	4.01 (2.56-6.26)	0.0000	54.64 (13.36-223.40)	0.0000
1969-45	*51-60*	454,686	72	15.84 (12.58-19.94)	2.78 (1.98-3.90)	0.0000	151.96 (37.28-619.33)	0.0000
<1945	*>60*	44,869	9	20.06 (10.55-38.12)	1.26 (0.63-2.53)	0.5027	192.48 (41.59-890.85)	0.0000

CI- confidence interval

Table S3. Spearman correlation (0>R>1 and p<0.05 trend increase, -1>R>0 and p<0.05 trend decrease) in age groups of seropositive donors.

Group of donors	Age group					
	≤20	21-30	31-40	41-50	51-60	>60
**FT and RP**	R=-0.99; p<0.05	R=-0.98; p<0.05	R=-0.96; p<0.05	R=-0.92; p<0.05	R=-0.74; p<0.05	R=-0.67; p<0.05
**FT**	R=-0.98; p<0.05	R=-0.96; p<0.05	R=-0.96; p<0.05	R=-0.27; p>0.05	R=+0.46; p>0.05	R=-0.29; p>0.05
**RP**	R=-0.59; p<0.05	R=-0.77; p<0.05	R=-0.81; p<0.05	R=-0.35; p>0.05	R=-0.57; p<0.05	R=+0.18; p>0.05

R - Spearman correlation coefficient. p - statistical significance level

Table S4. Frequency of seropositive HBV (HBsAg+) infections in donor age groups in total, in Poland, in 2018-2019.

Year of birth	Age group	No.		Frequency/10^5 (95% CI)	Relative Risk (RR) between age groups			
		tested	HBsAg(+)		RR (95% CI) *younger group as a reference group*	p	RR (95% CI) *youngest group as a reference group*	p
a) In total								
1998-2001	*18-20*	186,572	5	2.68 (1.14-6.27)	-	-	-	-
1988-1998	*21-30**	423,121	139	32.85 (27.83-38.78)	12.25 (5.02-29.91)	0.000	12.25 (5.02-29.91)	0.000
1978-1988	*31-40*	352,585	137	38.86 (32.87-45.93)	1.18 (0.93-1.49)	0.1628	14.49 (5.94-35.38)	0.000
1953-1978	*41->65**	262,497	110	41.91 (34.77-50.50)	1.07 (0.83-1.38)	0.5551	15.63 (6.38-38.31)	0.000

**In total*: RR calculated for 21-30 and 41->65 age group was 1.27 (0.99-1.62), p=0.0559

Year of birth	Age group	tested	HBsAg(+)	Frequency/10^5 (95% CI)	RR (95% CI) younger	p	RR (95% CI) youngest	p
b) First Time donors								
1998-2001	*18-20*	116,244	5	4.30 (1.84-10.07)	-	-	-	-
1988-1998	*21-30^*	91,670	138	150.54 (127.44-177.81)	34.99 (14.34-85.41)	0.000	34.99 (14.34-85.41)	0.000
1978-1988	*31-40*	54,323	137	252.20 (213.39-298.03)	1.67 (1.32-2.12)	0.000	58.63 (24.02-143.10)	0.000
1953-1978	*41->65^*	34,718	106	305.32 (252.52-369.11)	1.21 (0.93-1.55)	0.1395	70.98 (28.94-174.04)	0.000

^*First Time*: RR calculated for 21-30 and 41->65 age group was 2.02 (1.57-2.61), p=0.000

Year of birth	Age group	tested	HBsAg(+)	Frequency/10^5 (95% CI)
a) Repeat donors				
1998-2001	*18-20*	70,328	0	0.00 (0,00-5.46)
1988-1998	*21-30#*	331,451	1	0.30 (0.05-1.71)
1978-1988	*31-40*	298,262	0	0.00 (0.00-1.29)
1953-1978	*41->65#*	227,775	4	1.76 (0.68-4.52)

Repeat Donors: RR calculated for 21-30 a 41->65 age group was 5.82 (0.65-52.07), p=0.0739 NS

Figure S4. Changes of HBV seropositive infections frequency (/1000 donors) in age groups, in Poland, in the period 2005-2019 in FT and RP.

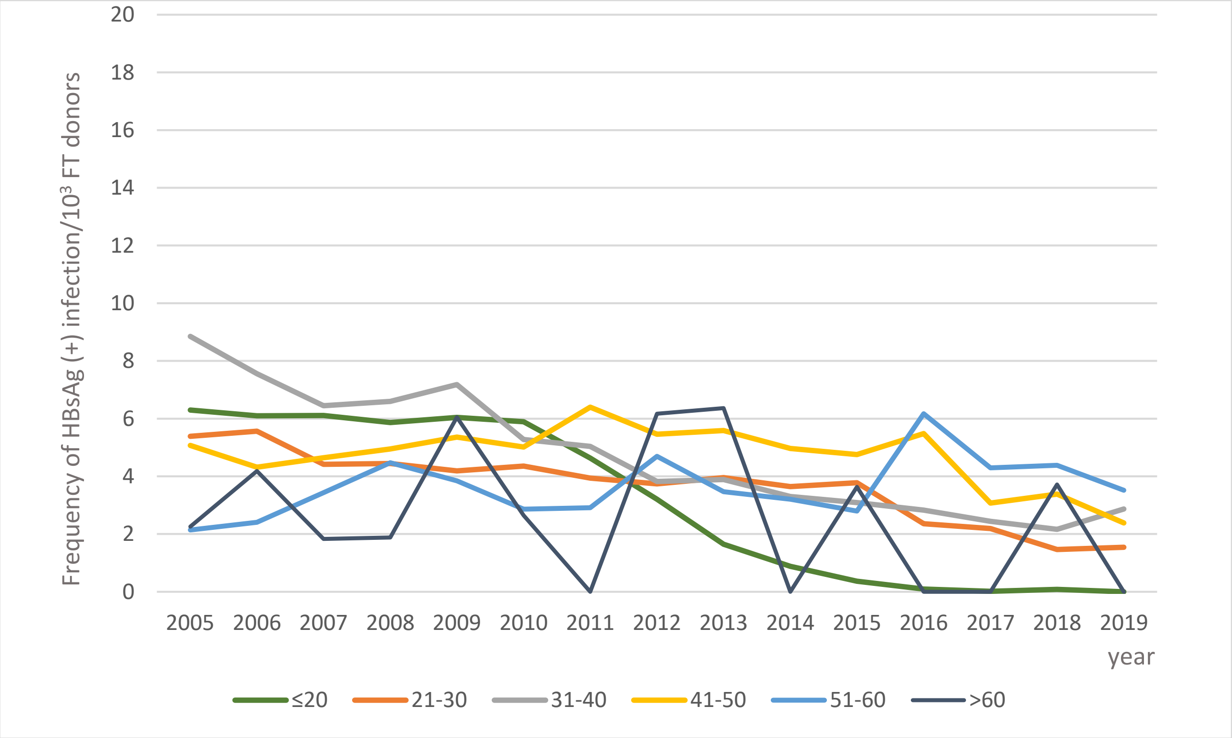

Figure S5. Change (%) in the age structure of FT donors [15].

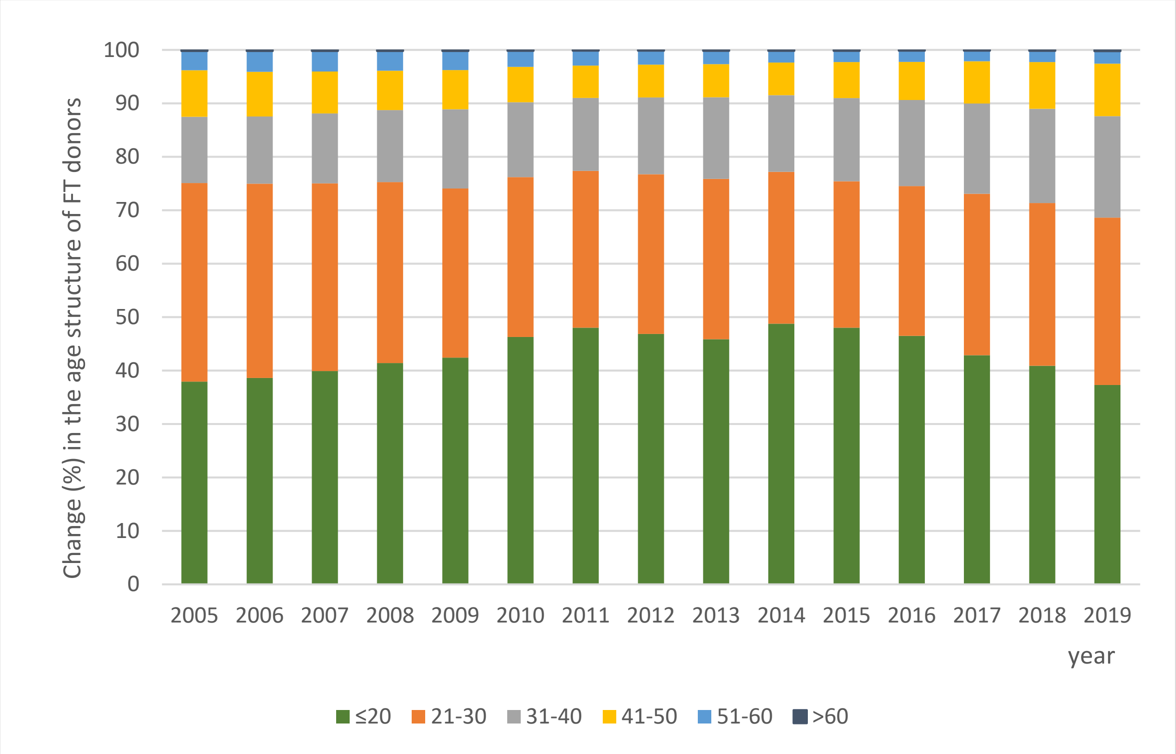

Figure S6. Fraction (%) of adult residents in Poland vaccinated as a newborn, changes in 2012-2019 [11].

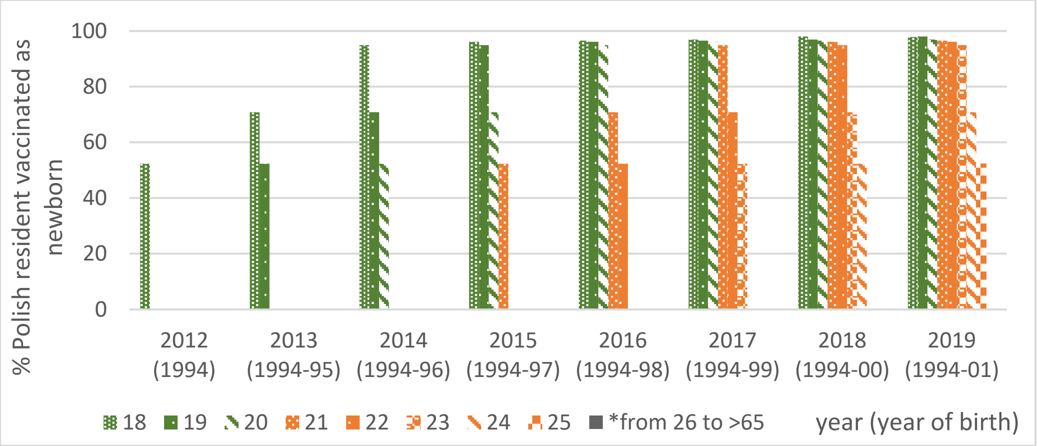

Figure S7. Fraction (%) of adult residents in Poland vaccinated as 14-year-old adolescence, changes in 2005-2019 [37].

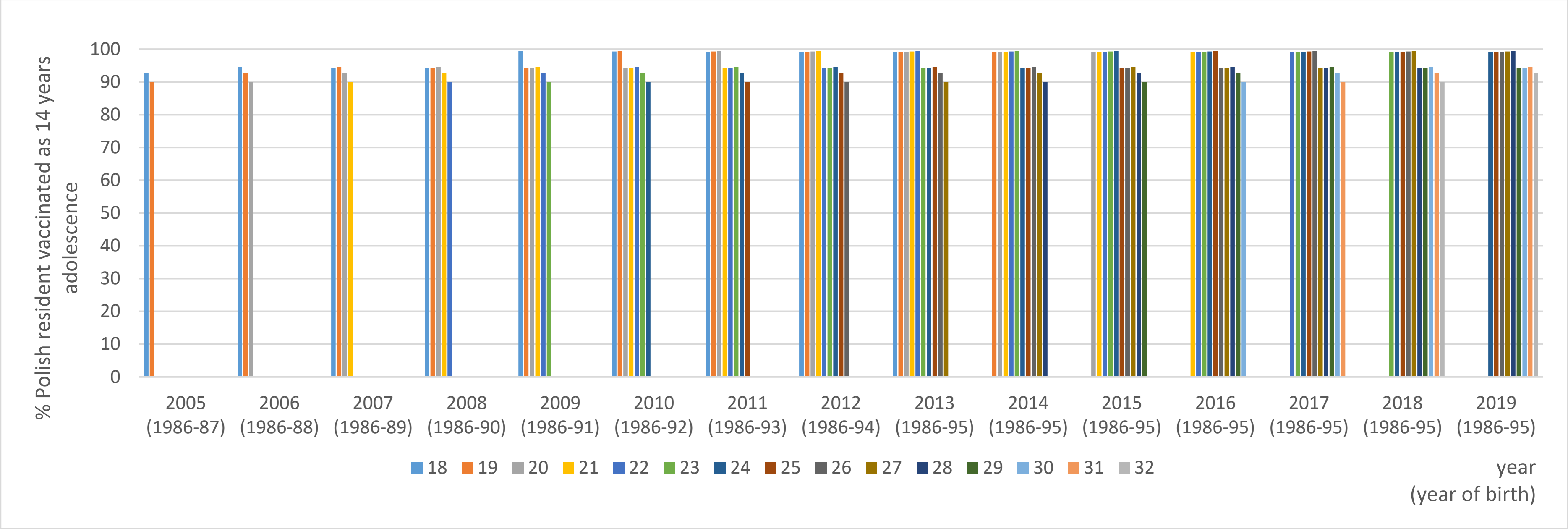

Supplement: Supplementary file 1 [file viruses-17-00060-s001.zip › viruses-3341476-supplementary.pdf]
